# Psychometric Properties of the Multidimensional Assessment of Interoceptive Awareness (MAIA) Questionnaire in Colombian University Students

**DOI:** 10.3390/jcm12082937

**Published:** 2023-04-18

**Authors:** Olga Montoya-Hurtado, Nicolás Gómez-Jaramillo, Gloria Bermúdez-Jaimes, Luis Correa-Ortiz, Sandra Cañón, Raúl Juárez-Vela, Iván Santolalla-Arnedo, Laura Criado-Pérez, Jesús Pérez, María Consuelo Sancho-Sánchez, José Criado-Gutiérrez

**Affiliations:** 1 Research Department, Escuela Colombiana de Rehabilitación, Health and Sports Sciences, Bogota 110121, Colombia; olga.montoya@ecr.edu.co (O.M.-H.);; 2 Postgraduate Unit, Program in Health, Disability, Dependency, and Well-Being, University of Salamanca, 37007 Salamanca, Spain; 3 Research Department, Universidad de Manizales, Engineering and Medicine, Manizales 170003, Colombia; 4 Department of Nursing, GRUPAC, University of La Rioja, 26004 Logroño, Spain; 5 Centro de Investigación Biomédica de La Rioja, 26006 Logroño, Spain; 6 Faculty of Medicine, Department of Surgery, University of Salamanca, 37007 Salamanca, Spain; lauracriado@usal.es; 7 Faculty of Medicine, University of Salamanca, 37007 Salamanca, Spain; 8 Department of Psychiatry, University of Cambridge, Cambridge CB2 0SL, UK; 9 Norwich Medical School, University of East Anglia, Norwich NR4 7TJ, UK; 10 Department of Physiology and Pharmacology, University of Salamanca, 37007 Salamanca, Spain; sanchoc@usal.es (M.C.S.-S.);; 11 Institute of Neurosciences of Castilla y León (INCYL), 37007 Salamanca, Spain

**Keywords:** awareness, interoception, evaluation study, students, psychometry

## Abstract

Introduction: The university student population is influenced by multiple factors that affect body awareness. Identifying the body awareness status of students is crucial in creating self-care and emotion management programs to prevent diseases and promote health. The Multidimensional Assessment of Interoceptive Awareness (MAIA) questionnaire evaluates interoceptive body awareness in eight dimensions through 32 questions. It is one of the few tools that enable a comprehensive assessment of interoceptive body awareness by involving eight dimensions of analysis. Method: The objective of this study is to present the psychometric properties of the Multidimensional Assessment of Interoceptive Awareness (MAIA) to observe to what extent the hypothesized model fits the population of university students in Colombia. A descriptive cross-sectional study was conducted with 202 students who met the inclusion criterion of being undergraduate university students. Data were collected in May 2022. Results: A descriptive analysis of the sociodemographic variables of age, gender, city, marital status, discipline, and history of chronic diseases was performed. JASP 0.16.4.0 statistical software was used to conduct confirmatory factor analysis. A confirmatory factor analysis was performed based on the proposed eight-factor model of the original MAIA, giving a significant *p*-value and 95% confidence interval. However, when performing loading factor analysis, a low *p*-value was found for item 6 of the Not Distracting factor, and for the entire Not Worrying factor. Discussion: A seven-factor model with modifications is proposed. Conclusions: The results of this study confirmed the validity and reliability of the MAIA in the Colombian university student population.

## 1. Introduction

The mental health of university students has been affected by various social determinants such as high academic load, a sedentary lifestyle, suicidal ideation, depression, early pregnancy, domestic violence, dysfunctional families, poverty, and eating disorders, among others [1]. This has generated interest by researchers in investigating these social determinants in greater depth to promote primary health care programs and generate a culture of care in universities [2].

Some prior studies promote health protective factors such as physical exercise and note that self-esteem is among the switch projective factors for protective factors. One of the pillars of this approach is to ensure adequate body awareness, which implies a conscious mind–body connection linked to internal processes of self-knowledge and self-regulation, confidence in the body, and identification of basic physical sensations such as postural alterations, respiratory and cardiac rhythm, in addition to identifying pain and states of relaxation [3].

Among the theoretical references to the body is corporeality, which refers to the understanding of the body beyond the physical, where an emotional memory produced by the interactions and intersections of the individual with a social context is registered throughout life [4]. Embodiment is related to the perceptual processes that give meaning, representation, and awareness to the body. These sensory and perceptual processes involve body image and body awareness. Body image has neurophysiological, psychological, and behavioral information that shapes the self-image that each person has of his or her own body [5]. Body awareness is the ability to identify the body’s signals to respond in time to situations that may affect health [6]. Body awareness requires information that the body receives from different sources. This information is processed at the neurophysiological level and converted into meanings, known as perceptions, which leave an imprint at the molecular level [7].

When individuals identify bodily sensations and their meaning, they are making themselves conscious of the internal information of their bodies, which is known as interoceptive body awareness. Body awareness can be affected by socioeconomic, cultural, and environmental conditions, and is rarely assessed in university students [4]. Identifying the body awareness status of students is important for creating self-care and emotion management programs. Wellness units are increasingly looking for tools that allow early identification of risks that may affect students’ health conditions, in order to implement promotion and prevention programs [3].

Interoceptive awareness refers to the ability to perceive and understand internal bodily signals, which is important for emotional regulation, decision-making, and stress adaptation. Evaluating interoceptive awareness requires sophisticated techniques and is typically conducted by specialists. Self-report questionnaires are an easy and cost-effective technique that can provide valuable information on how individuals perceive their bodily sensations, identify health issues, and track changes over time. However, self-report questionnaires may have limitations, such as potential for bias, lack of objectivity, and limited ability to measure the physiological aspects of interoceptive awareness. Nonetheless, self-report questionnaires remain a useful and accessible tool for evaluating interoceptive awareness [8,9]. 

Few tools exist to assess interoceptive body awareness in a multidimensional manner. A study conducted by two universities in the United Kingdom proposes a three-dimensional model that assesses interoceptive accuracy (performance in objective behavioral tests of beat detection, interoceptive sensitivity, and interoceptive awareness [10]. 

There are other tools to assess body awareness qualitatively, such as BARS; however, to use it, it is necessary to be a basal body awareness therapist [11]. These considerations served as the basis for the creation of the MAIA questionnaire, a multidimensional self-report instrument designed to measure interoceptive body awareness [12]. The MAIA is one of the few instruments that allows a comprehensive assessment of interoceptive body awareness by involving eight dimensions of analysis through 32 items. There is also a 37-item version and another for children aged 7 to 17 years. For this reason, it is being used in different countries and populations. It is currently free to use, and 28 translations have been made available on the website of the Osher Center for Integrative Medicine www.osher.ucsf.edu/maia (accesed 3 June 2022).

To date, studies have been conducted on the psychometric characteristics of the MAIA in different linguistic and sociocultural groups. Most studies have maintained the original eight-factor model. Some have found problems with the estimation of the Not Distracted and Not Worried subscales. Items 8 and 10 of the Not Worried subscales have been consistently problematic because of their low factor loadings or loading on other factors [13,14,15]. There are proposed six-factor versions, excluding the dimensions mentioned above, and other proposals that maintain the eight factors with item modifications.

The purpose of this paper is to present the psychometric properties of the Multidimensional Assessment of Interoceptive Awareness (MAIA) to observe to what extent the hypothesized model fits the population of university students in Colombia. Based on the studies taken as the background to our study, confirmatory analysis was applied with the model proposed by Chile, since this version was applied to a Spanish-speaking population; however, the model did not converge with the data from the Colombian student population; therefore, the original version of the MAIA was used.

Factor analyses are useful for researchers who apply instruments because they allow verification of the hypotheses of theoretical constructs, their validity, and reliability for application in specific populations. Confirmatory factor analysis allows the researcher to verify a questionnaire for use in different cultural contexts. The exploratory and confirmatory factor analysis of the original version of MAIA will be conducted to test the model in the Colombian university student population, replacing the previous analysis [16].

## 2. Materials and Methods

### 2.1. Sample and Validation Compliance with the Assumptions for the Application of Factor Analysis

The aim of this study is to present the psychometric properties of the Multidimensional Assessment of Interoceptive Awareness (MAIA) to observe to what extent the hypothesized model fits the population of university students in Colombia. The questionnaire was applied to a sample of 232 students, with a final sample of 202. Incomplete questionnaire data were excluded. According to Parra (2019), for sample calculation, 5 to 10 participants per item should be recruited [17]. For the MAIA questionnaire, which has 32 items, the minimum sample should be 160 participants. As a reference for this sample, a study was found that conducted a factor analysis of the psychometric properties of MAIA in a respondent sample of 204 Portuguese university students (52% female; M = 21.3, SD = 3.9 years), where MAIA version 2 was applied [18].

This study was conducted with undergraduate university students from the Escuela Colombiana de Rehabilitación in the city of Bogotá and the Universidad de Manizales in the city of Manizales. A cross-sectional study was used with convenience sampling. The sample consisted of 202 students who met the criterion of being undergraduate university students. Postgraduate students were not included due to the short time they remained in the institutions. The reference age presented in a mean of 21 years, as most of the sample fell within this range.

For the description of the psychometric characteristics of the MAIA questionnaire, both exploratory and confirmatory factor analyses were conducted The Kaiser–Meyer–Olkin measure of sampling adequacy and Bartlett’s test of sphericity were used to assess data factorability. Bartlett’s test of significant sphericity (*p* < 0.0001) and the KMO index < 0.50 indicate an adequate sample to support factor analysis and the correlation matrix determinant was 2.29 × 10^−10^. Based on the results obtained, it was possible to perform exploratory and confirmatory factor analyses [19,20]. In the EFA, multiple criteria were used to determine the number of factors to retain, such as the simplicity of the solution (factor loadings 0.30 and no cross-loadings), examination of eigenvalues > 1, and the interpretability of the factor structure [21]. Internal consistency reliability was determined by calculating Cronbach’s alpha coefficient. Construct validity was estimated following Terwee’s recommendations [22].

### 2.2. Instruments

All participants completed the Spanish version of the MAIA, using ArcGIS Survey123, a free-to-use Spanish version, which analyzes interoceptive body awareness in 8 categories through 32 questions as follows (Table 1).

The scale uses a Likert-type measurement scale from 0 (never) to 5 (always). It gives a total score for the level of body awareness and a dimensional assessment. For the dimensional assessment, it is important to note that questions 5, 6, 7, 8, and 9 are reverse scored [12].

Before answering the questionnaire, the ethical considerations of the study were explained through informed consent, and it was verified that all students who responded had no cognitive difficulties in understanding the questions.

For the present study, we took as background research prior studies on the psychometric characteristics of the 32-item version of the MAIA, in order to conduct our study in a population of Spanish-speaking university students (Table 2).

The original version of the MAIA validated in a Chilean population was used as a reference for the factorial analysis [14].

### 2.3. Statistical Analysis

A descriptive analysis was conducted on sociodemographic variables including age, gender, city, marital status, discipline, and history of chronic diseases. For the factor analysis, statistical software JASP 0.16.4.0 and Python were utilized.

## 3. Results

### 3.1. Participant Characteristics

Most of the participating students were women (64%), were studying BHASE disciplines (91%), and were aged between 18 and 49 years for 55% were under 21 years of age, with a median age of 21 and a SD o±3.48. 96%. Most were single (65%) and lived in the city of Bogota. Of the participants, 16% reported having a history of chronic diseases. The presence of these chronic diseases was investigated, and it was found that 32% of this sub-group of students reported a history of diseases such as diabetes and respiratory diseases. This information was included in the characterization of students to obtain a general health profile. These histories were not exclusion criteria for administering the questionnaire since MAIA, being a self-report questionnaire, aims to evaluate the perception of interoceptive awareness (Table 3).

### 3.2. Exploratory Factor Analysis (EFA)

The calculation of the eigenvalues was carried out, obtaining eight factors corresponding to the values higher than 1.0: 11.4377455, 2.90782465, 2.0218799, 1.54278935, 1.40393595, 1.35838162, 1.13057784, 1.02032593.

For these eigenvalues, the contribution rate of the variation and the cumulative contribution rate of the variation are calculated, obtaining the results shown in Table 4.

The factor load is analyzed, and no clearly defined factors are obtained, so a varimax rotation is applied; in addition, the questions are separated according to the factors and dimensions proposed by the MAIA questionnaire and the following values are obtained. Based on this analysis, a grouping of questions with high values into a single factor is not identified in the dimensions of Noticing, Not Distracting, Not Worrying and Attention Regulation (Table 5).

### 3.3. Confirmatory Factor Analysis

A confirmatory factor analysis was performed based on the proposed factor model of the original MAIA, giving a significant *p*-value, as shown in Table 6.

### 3.4. Factor Loadings

A confirmatory factor analysis of the original MAIA was performed, which resulted in a significant *p*-value and a 95% confidence interval. However, during the factor loading analysis, a low *p*-value was found for item 6 of the Not Distracting factor, and for the entire Not Worrying factor. This suggests that these elements may not fit well with the proposed model, and caution should be exercised when interpreting results related to these elements (Table 7 and Table 8).

Finally, a global Cronbach’s alpha (α) of 0.90 and an omega coefficient (Ω = 0.96) were found.

The study conducted among a Chilean student population is the closest to the Colombian population; however, when applying the adjusted six-factor model proposed in the Chilean study, it did not converge with the data from the Colombian student population. The factorial analysis performed in this study used the original version of MAIA translated into Spanish in the validation study conducted by Chile, where they found significant factor loadings for the eight factors and the best goodness-of-fit statistics with 30 items [14].

Overall, the results of the present study suggest that the version of the MAIA used is a useful and reliable tool for measuring interoceptive awareness in the studied population. However, further studies are needed to confirm the validity and reliability of the MAIA in different populations and transcultural contexts, as well as to explore possible adjustments to the proposed model.

## 4. Discussion

The objective of this study is to present the psychometric properties of the Multidimensional Assessment of Interoceptive Awareness (MAIA) Spanish version to observe to what extent the hypothesized model fits the population of university students in Colombia. The questionnaire was applied to a sample of 202 students. Among this sample, a global Cronbach’s alpha (α) of 0.90 was found. Other studies report a Cronbach’s alpha (α) of 0.90 was found in 202 students aged between 18 and 32 years. This result is similar to those of the MAIA in the German version, the Spanish version, and the Italian version [14,21,23]. The results reveal an internal consistency through the omega coefficient (Ω = 0.95), which is considered good. Among prior studies carried out using the MAIA, application of the omega coefficient is not found; however, this sample was used in similar studies supporting their findings with the global Cronbach’s alpha [27,28,29].

For the EFA of the present study, an eight-dimensional factorial structure was contemplated. Because no clearly defined factors were obtained, a varimax rotation was applied; additionally, the questions were separated according to the factors and dimensions proposed by the MAIA questionnaire. It was found that there was no group of questions with high values in a single factor in the dimensions relating to noticing, non-restlessness and regulation of attention. This can be coindexed with the study conducted in Chile with a sample of 470 participants aged between 18 and 70 years; the eight-factor EFA results found a model with loads greater than or equal to 0.30, where seven of the eight factors comprised three or more. The eight-factor model achieved the highest quality and was used to perform the AFC. As an analytical strategy, they used the ML method with Spanish Promax rotation [14].

A translation and validation study in Malaysia with 815 Malaysians (403 females) suggested a 19-item, three-factor structure. The confirmatory factor analysis indicated that both the three-factor and eight-factor models exhibited complete strict invariance between the sexes. Overall, the three-dimensional Malaysian MAIA proved to be internally consistent and invariant between the sexes, but further tests of construct and convergent validity are required [27]. A cross-sectional study involving 268 Japanese individuals proposed a six-factor structure that proved useful for assessing interoceptive awareness in the Japanese population [26]. In the confirmatory factorial analysis, the Japanese six-factor model showed a good fit to the original model [30]. The results suggest the need to make minor modifications, such as the elimination or addition of items to the original eight-factor model, to validate the MAIA scale in transcultural contexts.

We propose a seven-factor model with modifications, removing the Not Worrying factor, as it has a *p*-value of 0.0405, and item 6 of the Not Distracting factor, as it has a *p*-value of 0.087. Confirmatory factor analysis was performed with this proposal, giving the following results (Table 9).

**Figure 1:**
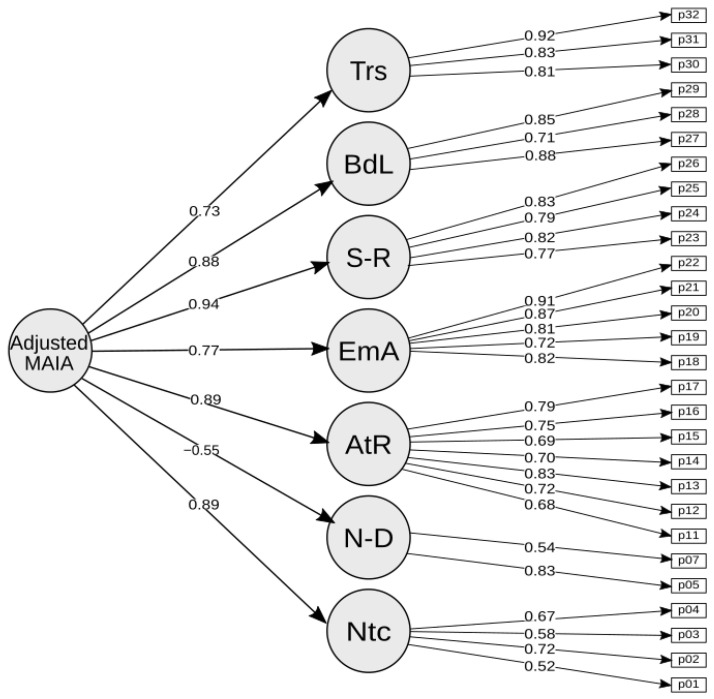
Proposed seven-factor MAIA.

A confirmatory factor analysis was performed based on the proposed factor model of the original MAIA, giving a significant *p*-value and a 95% confidence interval. However, the factorial loading analysis found a low *p*-value for item 6 of the Not Distracting factor, and for the entire Not Worrying factor (Table 10 and Figure 1).

This study showed that the Spanish version of the 32-item MAIA applied to the Colombian population has acceptable psychometric properties. The adjusted exploratory factor analysis suggested an eight-factor model; however, it is suggested to verify the dimension of Noticing, Not Worrying, and Attention Regulation. Some studies suggest models with six or seven factors by discarding some items [25]. 

## 5. Conclusions

This study showed that the Spanish version of the 32-item MAIA applied to Colombian university students has adequate psychometric properties in terms of validity and reliability. 

The CFA suggested a seven-factor model discarding the entire Not Worrying factor and item 6 of the Not Distracting factor.

The MAIA shows good overall internal consistency reliability and is a suitable instrument to assess interoceptive awareness in the population of university students with different sociodemographic characteristics.

It is important that the questionnaire is completed by university students who understand the questions.

## Figures and Tables

**Table 1:** MAIA categories and questions.

Categories	Questions
Noticing: Awareness of discomfort, comfort, and neutral bodily sensations	1, 2, 3, and 4
Not distracting: The tendency not to ignore or distract from the feeling of pain or discomfort	5, 6, and 7
Not Worrying: Tendency not to worry or to experience emotional stress with sensations of pain or discomfort	8, 9, and 10
Attention Regulation: The ability to sustain and control attention to bodily sensations.	11, 12, 13, 14, 15, 16, and 17
Emotional Awareness: Awareness of the connection between bodily sensations and emotional states.	18, 19, 20, 21, and 22
Self-Regulation: The ability to regulate tension/distress/grief through paying attention to bodily sensations	23, 24, 25, and 26
Body Listening: Actively listening to the body to clarify itself	27, 28, and 29
Trusting: Trusting that the body manifests itself safely and reliably	30, 31, and 32

**Table 2:** Prior studies.

Authors	Country	Language	N	Population	Proposal
Abbasi et al. (2018) [23]	Iran	Persian	225	University students	They preserve MAIA’s original structure. The results of this study confirmed the validity and reliability of MAIA in an Iranian student population.
Baranauskas et al. (2018) [24]	Lithuania	Lithuanian	386	Students (biomedical sciences, humanitarian sciences, physical sciences, social sciences, technological sciences, and arts)	They propose a six-factor structure with 25 items. They remove Not Distracting and Noticing before the AFC due to their low α.
Calı et al. (2015) [13]	Italy	Italian	321	Healthy Italian psychology students	They keep the eight-factor structure, proposing 29 items with modifications.
Fujino (2019) [25]	Japan	Japanese	268	University students	They propose a six-factor structure with 25 items. They remove Not Worrying and Self-Regulation.
Shoji et al. (2018) [26]	Japan	Japanese	390	University students	They propose a six-factor structure with 25 items. Not Worrying and Self-Regulation were eliminated.
Valenzuela-Moguillansky & Reyes-Reyes (2015) [14]	Chile	Spanish	470	Undergraduate and postgraduate students	They keep the eight-factor structure, proposing 30 items with modifications.

**Table 3:** Demographics of university students (n = 202).

		n (%)	Total
Age	<21	111 (55)	202 (100)
	>21	91 (45)	
Gender	Female	129 (64)	202 (100)
	Male	73 (36)	
City	Bogotá	131 (65)	202 (100)
	Manizales	49 (35)	
Discipline	STEM ^1^	18 (9)	202 (100)
	BHASE ^2^	184 (91)	
History of illness	Yes	32 (16)	202 (100)
	No	170 (84)	
Marital status	Single	195 (96)	202 (100)
	Other (e.g., married, divorced, widowed)	7 (6)	

^1^ STEM = Science, technology, engineering, and maths. ^2^ BHASE = Business, humanities, health, arts, social science, and education.

**Table 4:** Contribution rates.

Own Value	Contribution Rate of Change	Cumulative Contribution Rate of Change
11.437745	0.357430	0.357430
2.907825	0.090870	0.448299
2.021880	0.063184	0.511483
1.542789	0.048212	0.559695
1.403933	0.043873	0.603568
1.358381	0.042449	0.646017
1.130573	0.035330	0.681348
1.020317	0.031885	0.713233

**Table 5:** Factorial load by questions according to MAIA dimensions.

Categories	Questions	F1	F2	F3	F4	F5	F6	F7	F8
Noticing	1	0.20765	0.20801	0.06213	0.00794	0.28629	0.36322	0.12151	0.14692
	2	0.15165	0.41423	0.13721	0.06311	0.57072	0.31138	−0.02324	0.08675
	3	0.41711	0.18719	0.00063	0.20844	0.06006	0.30148	0.19902	−0.18076
	4	0.19250	0.27742	0.16634	0.15372	0.06429	0.61791	−0.09384	0.00947
Not-distracting	5	−0.02683	−0.38841	−0.10949	−0.11728	−0.43014	0.02032	0.18313	−0.25766
	6	−0.00949	0.10221	−0.04071	−0.00496	−0.11134	0.02300	0.34183	−0.06309
	7	0.02309	−0.16059	−0.04698	−0.06020	−0.75344	−0.03383	0.12199	0.07387
Not-Worrying	8	0.01621	−0.26117	−0.03121	0.05407	0.00082	−0.05790	0.74669	0.03557
	9	−0.07004	−0.33186	−0.27186	−0.00514	0.04148	−0.38124	0.14279	−0.11395
	10	0.03963	0.10493	0.28657	0.08421	0.36182	−0.02711	−0.34554	0.30034
Attention Regulation	11	0.41465	0.19321	0.22409	0.10943	0.04434	0.18181	0.14671	0.62515
	12	0.35956	0.34224	0.57887	−0.06166	−0.03649	0.14812	0.08199	0.12037
	13	0.43315	0.13321	0.41575	0.22066	0.14997	0.35878	−0.08962	0.20219
	14	0.59964	−0.02457	0.28371	0.25177	0.01257	0.21682	−0.10338	0.00979
	15	0.29328	0.24426	0.54247	0.11761	0.10276	0.16917	0.25450	−0.09232
	16	0.23837	0.02717	0.68363	0.29686	0.17319	0.16821	−0.00144	0.05666
	17	0.22039	0.26814	0.76019	0.14585	0.06409	0.04958	0.06044	0.13230
Emotional Awareness	18	0.14737	0.77073	0.17636	0.21597	0.07851	0.13014	0.04153	−0.01667
	19	0.02696	0.72694	0.19936	−0.00819	0.25174	0.10619	0.05363	0.14865
	20	0.12706	0.71654	0.15779	0.10719	0.20746	0.19411	0.02610	0.09597
	21	0.17741	0.42976	0.39198	0.32846	0.25808	0.28322	0.05093	−0.17304
	22	0.16554	0.62383	0.14844	0.44967	0.10698	0.33288	−0.03908	−0.07341
Self-Regulation	23	0.52981	0.39217	0.03837	0.29841	0.25592	0.10250	−0.17905	0.05633
	24	0.62503	0.16149	0.14513	0.26761	0.12209	0.25809	−0.07940	0.22598
	25	0.35136	0.26023	0.31367	0.52867	0.20211	−0.02938	−0.12081	0.10851
	26	0.70789	0.13777	0.25954	0.27707	0.18182	−0.05147	−0.20550	0.09154
Body Listening	27	0.39265	0.36877	0.48276	0.16569	0.13107	0.06454	0.03456	0.33023
	28	0.72666	0.08741	0.25868	0.00331	−0.12086	0.00182	0.12731	0.10964
	29	0.82190	0.02088	0.18365	0.24502	−0.02224	0.16511	0.19739	0.00822
Trusting	30	0.28326	0.12009	−0.02199	0.76045	0.04376	0.03490	−0.02812	0.19795
	31	0.21471	0.08494	0.17788	0.85530	0.01586	0.03045	0.12089	−0.08772
	32	0.18132	0.16839	0.30496	0.64734	0.07792	0.38204	−0.0894	0.03105

**Table 6:** Confirmatory factor analysis.

Model	Χ^2^	gl	*p*
Model base	19,968.750	496	
Model factor	1152.873	456	<0.001

**Table 7:** Factor loadings of CFA.

95% Confidence Interval				
Factor	Item	*p*	Lower	Upper
Noticing	1	<0.001	0.133	0.336
	2	<0.001	0.182	0.460
	3	<0.001	0.144	0.364
	4	<0.001	0.168	0.423
Not Distracting	5	<0.001	0.520	0.863
	6	0.087	−0.009	0.134
	7	<0.001	0.352	0.568
Not Worrying	8	0.404	−0.080	0.199
	9	0.403	−0.264	0.658
	10	0.402	−0.550	0.221
Attention Regulation	11	<0.001	0.241	0.350
	12	<0.001	0.258	0.372
	13	<0.001	0.293	0.419
	14	<0.001	0.244	0.353
	15	<0.001	0.249	0.358
	16	<0.001	0.268	0.383
	17	<0.001	0.283	0.404
Emotional Awareness	18	<0.001	0.470	0.560
	19	<0.001	0.412	0.496
	20	<0.001	0.464	0.555
	21	<0.001	0.497	0.595
	22	<0.001	0.523	0.619
Self-Regulation	23	<0.001	0.206	0.377
	24	<0.001	0.218	0.402
	25	<0.001	0.209	0.385
	26	<0.001	0.219	0.403
Body Listening	27	<0.001	0.320	0.520
	28	<0.001	0.256	0.407
	29	<0.001	0.309	0.496
Trusting	30	<0.001	0.499	0.613
	31	<0.001	0.517	0.625
	32	<0.001	0.573	0.706

**Table 8:** Factor loading.

Factor	*p*	Lower	Upper
Noticing	<0.001	1.116	2.905
Not Distracting	<0.001	−0.839	−0.497
Not Worrying	0.405	−8.143	3.289
Attention Regulation	<0.001	1.681	2.486
Emotional Awareness	<0.001	1.118	1.367
Self-Regulation	<0.001	1.686	3.218
Body Listening	<0.001	1.397	2.326
Trusting	<0.001	0.927	1.168

**Table 9:** Adjusted confirmatory factor analysis.

Model	Χ^2^	gl	*p*
Model base	18,943.294	378	
Model factor	899.884	343	<0.001

**Table 10:** Adjusted factor loading.

Factor	*p*	Lower	Upper
Noticing	<0.001	1.117	2.801
Not Distracting	<0.001	−0.829	−0.484
Attention Regulation	<0.001	1.623	2.340
Emotional Awareness	<0.001	1.097	1.339
Self-Regulation	<0.001	1.691	3.973
Body Listening	<0.001	1.397	2.338
Trusting	<0.001	0.947	1.203

## Data Availability

Generated Statement: No potentially identifiable human images or data is presented in this study.
